# Early Life Stress and Adversity in Children: Neuroendocrine Mechanisms, Epigenetic Regulation, and Lifespan Developmental Outcomes—A Narrative Review

**DOI:** 10.3390/children13060802

**Published:** 2026-06-10

**Authors:** Panagiotis Pipelias, Christina Kanaka-Gantenbein, Panagiota Pervanidou

**Affiliations:** 1 Postgraduate Program “The Science of Stress and Health Promotion”, School of Medicine, National and Kapodistrian University of Athens, 106 79 Athens, Greece; chriskan@med.uoa.gr (C.K.-G.); ppervanid@med.uoa.gr (P.P.); 2 Unit of Developmental and Behavioral Pediatrics, First Department of Pediatrics, School of Medicine, National and Kapodistrian University of Athens, “Aghia Sophia” Children’s Hospital, 115 27 Athens, Greece; 3 Division of Endocrinology, Metabolism and Diabetes, First Department of Pediatrics, School of Medicine, National and Kapodistrian University of Athens, “Aghia Sophia” Children’s Hospital, 115 27 Athens, Greece

**Keywords:** early life stress, adverse childhood experiences, HPA axis, epigenetics, DNA methylation, developmental programming, lifespan health

## Abstract

**Highlights:**

**What are the main findings?**

- Early life stress (ELS) has been associated with long-term neuroendocrine, immune, and epigenetic alterations that shape developmental trajectories across the lifespan.
- Dysregulation of the hypothalamic–pituitary–adrenal (HPA) axis, placental signaling pathways, and stress-related epigenetic mechanisms represent key mediators linking early adversity to later physical and mental health outcomes.

**What are the implications of the main findings?**

- Identification of stress-related neurobiological and epigenetic pathways may support earlier detection of vulnerable pediatric populations and improve preventive intervention strategies.
- Understanding developmental programming mechanisms may facilitate the development of targeted, multidisciplinary approaches to reduce the long-term burden of stress-related disorders.

**Abstract:**

Early life stress (ELS) and adverse childhood experiences are critical determinants of neurodevelopmental trajectories and long-term somatic and psychiatric health outcomes. This narrative review synthesizes current evidence, identified through searches in PubMed, Scopus, and Web of Science, on the neurobiological and epigenetic mechanisms through which early environmental exposures shape developmental programming and stress responsivity across the lifespan. A central framework is the dysregulation of the hypothalamic–pituitary–adrenal (HPA) axis, which mediates adaptive and maladaptive stress responses. During sensitive developmental periods, including prenatal, perinatal, and early postnatal stages, increased neuroplasticity confers heightened vulnerability to environmental influences, resulting in persistent alterations in stress regulation systems, brain circuitry, and endocrine function. The review further examines the role of maternal stress during gestation, with emphasis on placental regulatory mechanisms and fetal programming processes that establish long-term physiological set points. In parallel, emerging evidence on paternal stress is considered, highlighting potential contributions of germline epigenetic modifications and postnatal environmental transmission pathways. At the molecular level, epigenetic mechanisms—including DNA methylation, histone modifications, and non-coding RNA regulation—are discussed as key mediators linking early environmental exposures to stable changes in gene expression without alterations in DNA sequence. Collectively, the evidence supports ELS as a fundamental biological embedding process with enduring consequences for health across the lifespan. A deeper understanding of these mechanisms, alongside the identification of reliable biomarkers, is essential for early detection and the development of targeted preventive and intervention strategies in pediatric populations.

## 1. Introduction

Early life represents a critical period of heightened neurobiological plasticity during which environmental exposures can exert profound and long-lasting effects on developmental trajectories. Among these exposures, early life stress (ELS) and adverse childhood experiences (ACEs) have emerged as major determinants of both immediate and long-term health outcomes. A growing body of evidence indicates that exposure to stress during prenatal, perinatal, and early postnatal periods is associated with increased risk for a wide range of somatic, neurodevelopmental, and psychiatric disorders across the lifespan. Importantly, these effects are particularly relevant in pediatric populations, where early developmental processes are highly sensitive to environmental influences and may shape long-term health trajectories.

The concept of “biological embedding” provides a useful framework for understanding how early environmental experiences become integrated into physiological systems. Through this process, chronic or repeated activation of stress-responsive systems—particularly the hypothalamic–pituitary–adrenal (HPA) axis and the autonomic nervous system (ANS)—can lead to persistent alterations in neuroendocrine function, immune regulation, and brain development. These changes are especially pronounced during sensitive developmental windows when regulatory systems are still maturing and are therefore more susceptible to environmental modulation.

In parallel, advances in molecular biology have highlighted the role of epigenetic mechanisms as key mediators linking early environmental exposures to long-term changes in gene expression. Processes such as DNA methylation, histone modification, and non-coding RNA regulation allow environmental signals to influence biological systems without altering the underlying DNA sequence. These mechanisms provide a plausible pathway through which early life stress may exert enduring effects on stress responsivity, neurodevelopment, and disease vulnerability.

Despite substantial progress in this field, several important challenges remain. Findings across studies are often heterogeneous, reflecting differences in study design, timing and type of stress exposure, and methodological approaches. Furthermore, the predominance of observational and cross-sectional designs limits causal inference and complicates the identification of mechanistic pathways linking early stress exposure to long-term outcomes. In addition, the translation of findings from animal models to human populations remains complex, particularly in the context of developmental timing and environmental variability.

Given these considerations, a comprehensive and integrative synthesis of current evidence is needed, with particular emphasis on mechanisms relevant to child health and development. The present narrative review examines the neuroendocrine and epigenetic pathways through which ELS influences developmental programming and long-term health outcomes, with special attention to prenatal and early postnatal periods, as well as maternal and paternal contributions, and their clinical implications for early identification and intervention in pediatric populations, including improved screening, risk stratification, and preventive strategies.

Although previous reviews have examined specific aspects of ELS, including HPA axis dysregulation, prenatal stress exposure, or epigenetic programming, relatively few have integrated these mechanisms within a comprehensive developmental framework relevant to pediatric populations. The existing literature is often dispersed across individual biological domains, limiting a comprehensive understanding of how these systems interact during critical developmental periods.

The present narrative review aims to address this gap by bringing together key findings from maternal, paternal, placental, neuroendocrine, immune, microbiome-related, and epigenetic research, with the goal of providing an integrative developmental perspective on the biological embedding of early adversity. Particular emphasis is placed on the interaction between prenatal and postnatal influences and their cumulative impact on long-term health trajectories. Rather than providing a systematic or quantitative synthesis, this review offers a structured conceptual integration of the available evidence, highlighting shared and interacting biological pathways through which ELS may shape developmental and health outcomes across the lifespan.

## 2. Methodology of the Review

This narrative review was conducted through a comprehensive literature search of PubMed, Scopus, and Web of Science databases. The literature search was performed between January and April 2026 and focused primarily on studies published between 2000 and 2026. Search terms included combinations of the keywords “early life stress”, “adverse childhood experiences”, “childhood trauma”, “HPA axis”, “epigenetics”, “DNA methylation”, “prenatal stress”, “maternal stress”, “paternal stress”, “placenta”, “developmental programming”, and “neurodevelopment”.

Priority was given to systematic reviews, meta-analyses, longitudinal cohort studies, and landmark experimental and clinical investigations examining the neuroendocrine, epigenetic, developmental, and health-related consequences of ELS. Additional relevant studies were identified through manual screening of reference lists from key publications.

Studies were selected based on their relevance to the objectives of this review, namely the biological mechanisms linking early adversity with developmental programming and lifelong health outcomes. Articles not directly related to ELS, developmental stress biology, or epigenetic regulation were excluded. Non-English publications and abstracts without available full-text articles were also excluded. The narrative review aimed to provide a comprehensive and integrative synthesis of current evidence across neuroendocrine, developmental, and epigenetic domains.

For the purpose of synthesis, the identified literature was organized into predefined thematic categories: (i) early life stress and childhood trauma, (ii) neuroendocrinology of early life stress, (iii) effects of early life stress, (iv) maternal and paternal stress, and (v) epigenetic mechanisms. This thematic organization was adopted primarily to facilitate conceptual clarity and systematic presentation of a complex and multidimensional field. It should be noted that this classification was used solely for organizational purposes and to facilitate discussion of the available evidence. In reality, the biological and environmental factors associated with ELS are closely interrelated and often influence one another. Neuroendocrine, immune, placental, developmental, and epigenetic processes interact throughout development, contributing collectively to developmental programming and long-term health outcomes. Accordingly, the thematic categories presented in this review should not be interpreted as independent mechanisms but rather as complementary components of a broader and interconnected developmental framework.

## 3. Stress: Definition and Neuroendocrine Regulation

Stress is conceptualized as a dynamic adaptive process through which the organism responds to internal or external challenges that threaten homeostasis [1]. Rather than representing a simple reaction, stress reflects an integrated regulatory state that enables physiological and behavioral adaptation to environmental demands. Stressors may be physical or psychological and may be real or perceived [2]. Central to this process is the maintenance of homeostasis through the coordinated activation of neuroendocrine and autonomic systems. When homeostatic balance is challenged, the organism engages a highly conserved stress response system that integrates central nervous system (CNS) processing with peripheral effector mechanisms [3].

The stress response is primarily mediated by two interacting systems: the hypothalamic–pituitary–adrenal (HPA) axis and the autonomic nervous system (ANS). Within the CNS, stress-related information is integrated through limbic, cortical, and brainstem circuits, which coordinate endocrine and autonomic outputs [4]. Activation of the HPA axis is initiated by the hypothalamic paraventricular nucleus (PVN) through the release of corticotropin-releasing hormone (CRH) and arginine vasopressin (AVP). This leads to adrenocorticotropic hormone (ACTH) secretion from the anterior pituitary and subsequent glucocorticoid release from the adrenal cortex. In parallel, activation of brainstem noradrenergic pathways, particularly the locus coeruleus (LC), triggers sympathetic nervous system output and catecholamine release, primarily epinephrine and norepinephrine [5]. Glucocorticoids (GCs), primarily cortisol in humans, act through intracellular glucocorticoid and mineralocorticoid receptors that function as transcriptional regulators of gene expression. Through these mechanisms, they influence immune function, metabolism, and neural plasticity, thereby shaping both acute adaptive responses and long-term physiological regulation [6]. The ANS complements HPA axis activity through its sympathetic and parasympathetic branches, which exert opposing but coordinated effects to ensure rapid mobilization during stress and recovery following its resolution. Together, these systems form a tightly regulated network that enables adaptation to acute stressors while maintaining physiological stability [7].

Under conditions of chronic or excessive activation, this regulatory system may become dysregulated, leading to sustained alterations in stress responsivity. Such dysregulation contributes to allostatic load, a state of cumulative physiological burden associated with adverse effects on immune and metabolic function, as well as behavior [8]. Importantly, the stress system operates as a central interface between environmental experience and biological regulation, linking external challenges to long-term physiological and behavioral outcomes [9].

Importantly, the stress system undergoes significant maturation during early development, with regulatory mechanisms not yet fully established in infancy and early childhood. This developmental immaturity renders the stress system particularly sensitive to environmental inputs, contributing to the long-term calibration of stress responsivity. Despite extensive characterization of the stress response, measuring stress remains challenging due to discrepancies between subjective perception and biological markers. This complexity complicates the interpretation of stress-related findings, particularly in pediatric populations.

## 4. Early Life Stress and Childhood Trauma

ELS refers to a broad spectrum of adverse and highly stressful experiences occurring during prenatal, perinatal, childhood, or adolescent development that may disrupt normal developmental trajectories and exert long-term effects on physical health, psychological functioning, cognition, and behavior [10]. These experiences interfere with normative developmental programming and may alter the trajectory of brain maturation during critical and sensitive periods of development [11]. ELS encompasses a wide range of exposures during both the prenatal and postnatal periods, including maternal malnutrition, psychosocial stress during pregnancy, environmental adversity, and exposure to acute or chronic stressors such as violence, war, or natural disasters. In postnatal life, ELS includes experiences such as neglect, abuse, parental loss, poverty, household dysfunction, and exposure to community or domestic violence [12].

Within this framework, childhood trauma represents a more specific and severe subset of ELS, typically involving experiences that are emotionally overwhelming, life-threatening, or associated with actual or perceived threat to physical integrity. These include physical, emotional, or sexual abuse, severe neglect, traumatic loss, and exposure to extreme violence or disaster. Trauma is characterized not only by the nature of the event but also by the individual’s perceived inability to cope at the time of exposure [13]. A widely used conceptualization of cumulative adversity is provided by adverse childhood experiences (ACEs), which include domains such as abuse, neglect, and household dysfunction (e.g., parental mental illness, substance abuse, incarceration, or family disruption) [14]. Importantly, ACEs are strongly associated with dose-dependent increases in risk for a wide range of psychiatric and somatic disorders [10]. However, the relationship between ACEs and health outcomes is not always linear, as threshold effects and resilience factors may modify individual risk trajectories. A critical distinction exists between single traumatic events and complex or chronic trauma, with the latter being defined by repeated or prolonged exposure to adverse experiences during key developmental periods. Such chronic exposure is particularly detrimental due to its interaction with ongoing neurodevelopmental processes, thereby increasing the risk of long-term dysregulation across stress system and cognitive functioning [15].

From a public health perspective, ELS and childhood trauma represent a major global burden, affecting a substantial proportion of the population and contributing significantly to morbidity, mortality, and healthcare costs [16]. Epidemiological evidence indicates that exposure to multiple ACEs is associated with reduced life expectancy and increased risk for chronic conditions, including cardiovascular, metabolic, respiratory, and psychiatric disorders [17,18,19]. Collectively, ELS is increasingly recognized as a major developmental risk factor that operates through neurobiological embedding mechanisms, linking early environmental adversity with long-term alterations in stress regulation and health trajectories across the lifespan.

## 5. Neuroendocrinology of Early Life Stress

**Figure 1:**
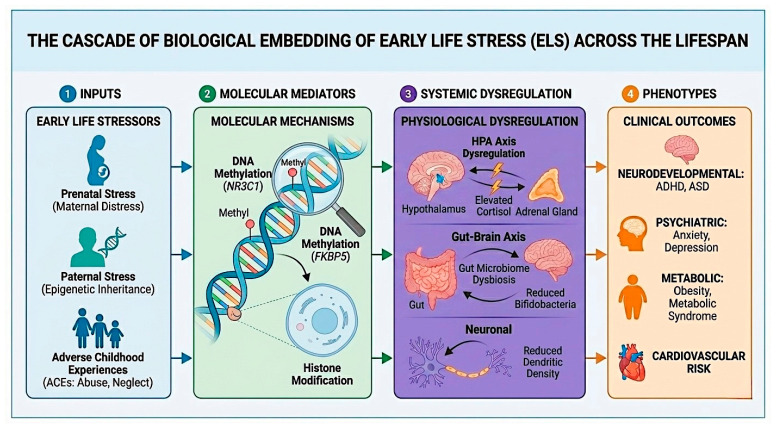
The multi-level cascade of biological embedding of early life stress (ELS).

ELS has been associated with long-lasting effects on multiple neurobiological systems involved in stress regulation, development, and physiological adaptation. Both clinical and preclinical evidence indicate that early adverse experiences can induce persistent alterations in neuroendocrine function, which may contribute to long-term changes in stress responsivity and increased vulnerability to physical and mental disorders (Figure 1).

### 5.1. HPA Axis and Glucocorticoid Programming

The early life environment plays a critical role in shaping the development and long-term regulation of the HPA axis. Exposure to parental adversity and elevated GCs, particularly during pregnancy, has been consistently associated with alterations in offspring stress responsivity. The impact of early stress on HPA axis function depends on multiple factors, including the timing of exposure, type and severity of the stressor, maternal stress perception, and offspring sex [20]. Notably, early gestation exposure appears to be particularly strongly associated with programming effects [21].

ELS has been associated with both the hyperactivation and hypoactivation of the HPA axis. Findings include increased basal cortisol levels, enhanced cortisol awakening response, elevated dehydroepiandrosterone (DHEA) concentrations and heightened ACTH reactivity, as well as blunted cortisol responses to psychosocial stressors [22,23] (Table 1). This variability likely reflects differences in developmental timing, chronicity, and cumulative stress exposure. Additionally, inconsistencies across studies may arise from differences in cortisol sampling methods (e.g., salivary vs. plasma), timing of assessment, and variability in stress paradigms, limiting the direct comparability of findings. A key determinant of these effects is the timing of exposure relative to sensitive developmental windows. Early postnatal life, particularly the first two years, represents a period of heightened plasticity, during which the HPA axis undergoes dynamic changes in stress responsivity. Disruption during this period may lead to long-term alterations in glucocorticoid signaling and feedback sensitivity [24].

At the molecular level, GCs act through glucocorticoid (GR) and mineralocorticoid (MR) receptors, particularly in limbic regions such as the hippocampus. Evidence from both animal and human studies suggests that excess early glucocorticoid exposure may be associated with reduced receptor expression and altered HPA axis feedback regulation. However, the underlying molecular mechanisms have been more extensively characterized in animal models than in human populations [25].

Finally, synthetic glucocorticoid exposure during pregnancy, although clinically necessary in certain conditions, has been linked to long-term alterations in offspring neuroendocrine function [26]. In addition to early childhood, adolescence represents another sensitive period during which stress exposure may further modify HPA axis responsivity [27]. Longitudinal evidence suggests that HPA axis alterations are not static but may shift across development, reflecting dynamic interactions between biological programming and ongoing environmental exposures.

Collectively, the literature indicates that ELS does not lead to a uniform pattern of HPA axis dysregulation. Rather, stress-related neuroendocrine outcomes appear to vary according to developmental timing, chronicity and severity of exposure, sex-specific factors, age at assessment, and methodological differences across studies. This heterogeneity likely contributes to the coexistence of both hyperactivation and hypoactivation profiles reported in the literature.

### 5.2. ANS Dysregulation

In addition to HPA axis alterations, ELS has been associated with ANS dysregulation, including increased sympathetic activity and reduced parasympathetic (vagal) tone [25]. This imbalance is reflected in increased catecholaminergic activity and reduced cardiovascular vagal regulation. These changes may contribute to heightened physiological arousal, altered stress responsivity, and increased vulnerability to stress-related psychopathology [28]. Early adverse experiences have also been linked to alterations in autonomic reactivity patterns, which may persist into adolescence and adulthood and contribute to long-term psychophysiological dysregulation [29]. Heart rate variability (HRV) has emerged as a non-invasive biomarker of autonomic regulation and may provide clinically relevant insights into stress-related dysregulation in children. However, standardization across studies remains limited.

### 5.3. Hypothalamic–Pituitary–Thyroid (HPT) Axis Interaction

The stress response involves interaction between the HPA and HPT axes, whose final effectors—GCs and thyroid hormones—are critical for fetal brain development, including neuronal proliferation, migration, and cortical maturation [30]. The HPT axis is regulated by thyrotropin-releasing hormone (TRH) from the hypothalamus and thyroid-stimulating hormone (TSH) from the pituitary [31]. Thyroid hormones act through nuclear receptors that are detectable in the developing brain from early gestation, while fetal thyroid function remains immature, making early neurodevelopment largely dependent on maternal thyroid hormone supply [32]. ELS and elevated GCs may suppress HPT axis activity, reducing maternal thyroid hormone availability and limiting fetal exposure to thyroxine [33]. This can adversely affect brain regions such as the cortex, hippocampus, and cerebellum during critical developmental windows [34]. Importantly, the HPA and HPT axes are bidirectionally linked, with stress hormones inhibiting thyroid function and thyroid hormones modulating stress system activity [2], highlighting their integrated role in neurodevelopmental regulation.

### 5.4. Monoaminergic Systems: Serotonergic and Dopaminergic Pathways

ELS also affects central monoaminergic systems, including serotonergic and dopaminergic pathways, which are involved in emotional regulation, reward processing, and cognitive development. During gestation, serotonin functions as a neurotrophic factor regulating neuronal proliferation, differentiation, and synaptogenesis [35]. Alterations in serotonergic signaling during early development may therefore have long-term effects on brain organization and behavioral outcomes [36]. Much of the mechanistic evidence supporting these effects originates from experimental animal studies, and direct extrapolation to human neurodevelopment should therefore be made with caution. Similarly, dopaminergic system alterations have been associated with changes in reward sensitivity and motivational processing following early adverse experiences. These effects may be mediated in part by glucocorticoid-induced changes in placental and fetal monoamine regulation [37].

### 5.5. Oxytocin and Stress Buffering Systems

The oxytocin system plays a key role in social bonding, attachment formation, and stress buffering. Oxytocin interacts with the HPA axis and can attenuate stress responses through the modulation of glucocorticoid activity. Early caregiving experiences significantly influence the development of oxytocin-mediated pathways. High-quality maternal care is associated with enhanced stress regulation and improved emotional resilience, whereas early maternal deprivation may exacerbate stress system dysregulation [38]. Although supported by both human and animal studies, many of the underlying neurobiological mechanisms have been primarily elucidated in animal models. Importantly, oxytocinergic mechanisms represent a critical biological pathway through which early attachment experiences can moderate the effects of ELS on neurodevelopmental outcomes [39]. Through these interacting systems, early environmental adversity becomes biologically embedded, shaping stress responsivity and developmental trajectories.

## 6. Effects of Early Life Stress

ELS, particularly during sensitive developmental windows, has been consistently associated with long-lasting alterations in brain structure and function, as well as increased vulnerability to physical and mental health disorders across the lifespan [40]. The magnitude and direction of these effects depend on the developmental timing of exposure, sex-specific vulnerability, and the age at outcome assessment [10].

### 6.1. Physical and Neuropsychiatric Outcomes

Exposure to ELS has been linked to a broad spectrum of somatic and psychiatric conditions. At the somatic level, associations have been reported with cardiometabolic disorders, including hypertension, type 2 diabetes, insulin resistance, obesity, and dysregulated metabolic profiles [41,42,43,44]. Additional evidence links ELS to respiratory dysfunction, atopic disease, and immune-related disorders [45,46,47]. In terms of mental health, ELS significantly increases the risk of depression, anxiety disorders, schizophrenia-spectrum conditions, antisocial behavior, and suicidal behavior [48,49,50,51,52]. Epidemiological estimates suggest that ELS may account for approximately 10–15% of the population-attributable risk for psychiatric disorders [53]. Furthermore, prenatal stress exposure has been associated with adverse perinatal outcomes, including low birth weight, intrauterine growth restriction, and pre-term birth [54,55].

### 6.2. Epigenetic and Cellular Aging Effects (Telomeres)

Prenatal and ELS have been associated with accelerated cellular aging, indexed by shorter leukocyte telomere length at birth and later life [56,57]. Telomeres serve as protective chromosomal caps, and their shortening reflects cumulative cellular stress and replicative aging. Mechanistically, stress-related telomere erosion is mediated by glucocorticoid signaling, oxidative stress, and inflammatory pathways (e.g., IL-6, TNF-α, CRP), which impair telomerase activity and promote DNA damage preferentially at telomeric regions [58]. These processes may represent a shared biological pathway linking early adversity with long-term disease susceptibility. However, findings on telomere length remain inconsistent, partly due to methodological variability and the predominance of cross-sectional designs, limiting causal inference.

### 6.3. Neurodevelopmental Disorders

ELS has been consistently associated with increased risk for neurodevelopmental disorders, including autism spectrum disorder (ASD) and attention-deficit/hyperactivity disorder (ADHD) [59,60]. Importantly, these associations may be influenced primarily by genetic predisposition as well as socioeconomic factors, complicating the interpretation of causal relationships. Proposed mechanisms include HPA axis dysregulation, placental signaling alterations, and disruptions in dopaminergic and serotonergic neurodevelopmental pathways [61]. According to Folger et al., each traumatic experience is associated with an 18% increase in the risk of developmental delay [62]. Children and adolescents exposed to complex trauma often exhibit aggression and atypical self-regulation. They frequently demonstrate impairments in the accurate processing of sensory information or may misinterpret sensory inputs [13], alongside significant difficulties in emotion regulation and in modulating levels of physiological and emotional arousal [63]. Also, prenatal stress has been shown to significantly affect the cognitive development of the child [64].

### 6.4. Post-Traumatic Stress Disorder

Prenatal stress exposure has been implicated in increased vulnerability to PTSD, both through neuroendocrine programming and postnatal caregiving interactions [65,66]. Altered cortisol rhythms and stress reactivity profiles are frequently observed in affected offspring [67].

### 6.5. Immune System and Gut Microbiome

Maternal stress during pregnancy is associated with altered maternal–fetal immune signaling, characterized by increased pro-inflammatory cytokines (IL-1β, IL-6, TNF-α) and reduced anti-inflammatory mediators such as IL-10 [68,69]. These changes may contribute to long-term immune dysregulation in offspring. In parallel, early stress exposure has been associated with alterations in gut microbiome composition, particularly during the first months of life. Reduced abundance of beneficial taxa (e.g., Bifidobacteria and Lactobacilli) and increased Proteobacteria have been reported, with implications for gastrointestinal function, allergic disease risk, and neurodevelopment [70,71,72]. These findings are consistent with the existence of a microbiota–gut–brain pathway that may contribute to the association between prenatal stress and long-term developmental outcomes, although causal relationships remain incompletely established in human populations. Although these findings are promising, most human studies remain observational and associative, limiting causal inference. Reverse causation and bidirectional interactions between stress physiology, immune function, environmental exposures, and gut microbial composition cannot be excluded. Consequently, the causal pathways linking early stress, microbiome alterations, and neurodevelopment remain incompletely understood and require further longitudinal investigation.

### 6.6. Circadian Rhythms and Sleep Regulation

The stress system and circadian timing system are bidirectionally interconnected. Early life adversity can disrupt circadian regulation, which may contribute to persistent alterations in sleep–wake patterns and chronobiological instability, which are commonly observed in trauma-related psychopathology [73,74].

### 6.7. Addiction and Reward-Related Behavior

Early adverse experiences are strong predictors of substance use disorders in adolescence and adulthood [75,76]. These effects are mediated by long-term alterations in mesolimbic dopamine circuitry, resulting in impaired reward sensitivity, increased impulsivity, and altered reinforcement learning processes [77,78]. Dysregulation of serotonergic and dopaminergic systems further contributes to increased vulnerability to addictive and externalizing behaviors [79].

### 6.8. Neuroimaging Evidence

Prenatal exposure to stress hormones has been associated with structural and functional alterations in the developing CNS. Dysregulation of circulating GCs may interfere with neurodevelopment through mechanisms such as neuronal loss, delayed myelination, and altered synaptic pruning [10]. These effects primarily involve stress-related brain circuits, including the prefrontal cortex, hippocampus, and amygdala. Neuroimaging studies have reported reductions in total brain volume, hippocampal volume [80], and cortical thickness in prefrontal and temporal regions, alongside decreased gray matter density [81]. In adults with a history of childhood trauma, reduced gray matter has been specifically observed in the right dorsolateral prefrontal cortex and hippocampus [82], suggesting long-term structural vulnerability. The hippocampus is particularly sensitive due to its high density of GR and prolonged postnatal development, continuing until approximately 2 years of age. The amygdala, which matures later in childhood and adolescence, is also highly susceptible to early stress exposure. Animal studies further indicate that prenatal stress increases dendritic complexity in the hippocampus and prefrontal cortex while reducing hippocampal neurogenesis, which are changes associated with impaired emotional regulation, heightened fear responsivity, and cognitive deficits [83]. While animal models provide valuable mechanistic insights, species-specific differences in brain maturation and developmental timing may limit direct translation of these findings to humans. Altered hippocampal function has also been linked to excessive in utero glucocorticoid exposure and disrupted HPA axis regulation [84]. Overall, early stress exposure appears to differentially affect key limbic and prefrontal circuits during critical windows of neurodevelopment, contributing to long-term alterations in emotional and cognitive functioning.

Despite the growing body of neuroimaging evidence linking ELS to structural and functional brain alterations, several methodological limitations should be considered when interpreting these findings. Many studies are based on relatively small sample sizes, which may reduce statistical power and increase the likelihood of inconsistent results. Considerable heterogeneity also exists regarding the type, timing, severity, and duration of stress exposure, as well as participant age, sex distribution, and socioeconomic background. Furthermore, variability in neuroimaging methodologies, image-processing approaches, and outcome measures complicates direct comparisons across studies. Importantly, the majority of available studies employ cross-sectional designs, limiting the ability to establish causal relationships between early adversity and later neurobiological alterations. Future large-scale longitudinal studies integrating neuroimaging, neuroendocrine, and environmental data are needed to clarify developmental trajectories and identify mechanisms underlying stress-related brain changes across the lifespan.

### 6.9. Resilience and Adaptive Programming

Despite the well-documented adverse effects of ELS, not all individuals exposed to early adversity develop negative outcomes. Protective factors, including supportive caregiving environments, stable social contexts, and individual differences in stress sensitivity, may promote resilience [85]. The concept of differential susceptibility suggests that some individuals are more responsive to both adverse and supportive environments, highlighting the importance of early intervention and environmental context [16].

The biological consequences of ELS arise through multiple interacting mechanisms involving neuroendocrine, immune, developmental, metabolic, and epigenetic pathways. The associations presented in Table 2 are intended to illustrate representative mechanisms and outcomes commonly reported in the literature. They should not be interpreted as exclusive or direct causal relationships, as substantial overlap and interaction exist among biological systems. Furthermore, the strength of evidence supporting individual pathways varies according to the outcome studied and the available human and experimental data.

These diverse outcomes are thought to share common underlying mechanisms, including chronic low-grade inflammation, neuroendocrine dysregulation, and altered stress responsivity.

## 7. Maternal Stress

### 7.1. Pregnancy and Early Life Experiences

Pregnancy constitutes a major biopsychosocial transition frequently accompanied by elevated stress levels, particularly in women with vulnerability to anxiety or depression. Approximately 75% of pregnant women report some degree of stress [86]. Expectant mothers often face concurrent social roles and both major and minor life stressors [87]. Around 8–12% meet criteria for a psychiatric disorder during pregnancy, while PTSD affects approximately 3.3% prenatally and 4% postpartum [88].

Prenatal maternal PTSD has been associated with obstetric complications, including abnormal fetal growth, miscarriage, hyperemesis, and pre-term birth. Postnatal PTSD has been linked to poorer infant weight gain and lower rates of breastfeeding. Importantly, prenatal PTSD significantly increases the risk of pre-term delivery, particularly when comorbid with depression [89]. Socioeconomic disadvantage further increases exposure to stressors, while social support may buffer adverse effects [90,91].

Early life experiences, both prenatal and postnatal, exert long-term effects on developmental trajectories through biological embedding mechanisms [16]. Neonatal HPA axis activity is initially immature, transitioning within the first months of life toward circadian regulation. Early caregiving plays a key role in shaping stress responsivity, with early hyporesponsivity potentially serving a protective developmental function [92].

Maternal cortisol levels during pregnancy are associated with infant emotional reactivity, altered stress physiology, and neurodevelopmental changes. Early gestational stress is linked to long-term HPA axis alterations and flattened cortisol rhythms in offspring [93]. Prenatal cortisol exposure also influences fetal gene expression and stress-related neurodevelopmental pathways [94]. Maternal metabolic, nutritional, and environmental exposures may induce epigenetic modifications with lasting effects on disease susceptibility [95,96]. These findings underscore the importance of early screening and intervention strategies targeting maternal mental health during pregnancy, which may have significant downstream effects on offspring development.

### 7.2. Placenta

**Figure 2:**
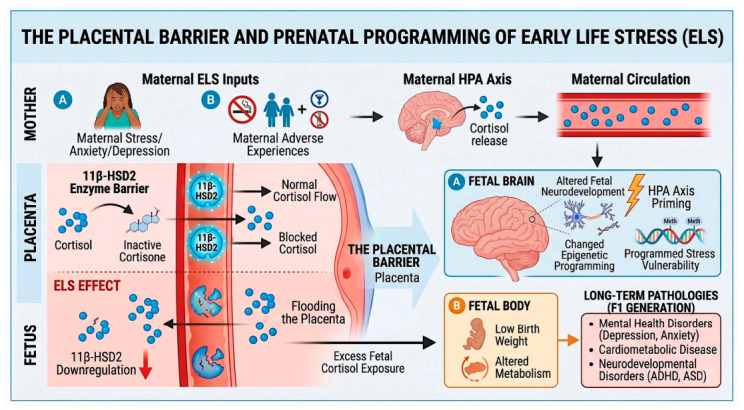
The placental barrier and prenatal programming of early life stress (ELS).

The placenta is a critical endocrine organ mediating maternal–fetal exchange and regulating fetal development. It produces CRH, which modulates both maternal and fetal HPA axis activity. Maternal stress increases placental CRH and glucocorticoid production, with fetal exposure occurring via partial placental transfer of cortisol [97,98]. GCs influence placental endocrine function, including prostaglandins, progesterone, and nutrient transport systems, thereby affecting fetal growth and neurodevelopment. Placental CRH rises progressively across gestation and contributes to the timing of parturition, with dysregulation associated with both pre-term and post-term birth [99,100]. Placental 11β-hydroxysteroid dehydrogenase type 2 (11β-HSD2) acts as a protective barrier by converting cortisol to inactive cortisone. Reduced enzyme activity, observed under maternal stress or depression, increases fetal glucocorticoid exposure and is associated with fetal growth restriction and preeclampsia [101,102,103,104]. Stress-related catecholamine release may further impair placental perfusion, promoting hypoxia, oxidative stress, and inflammatory activation, thereby disrupting neurodevelopmental processes such as neuronal migration and myelination [105]. Placental biomarkers, including CRH and 11β-HSD2 activity, may represent potential early indicators of fetal exposure to stress, although their clinical application remains under investigation (Figure 2).

### 7.3. Fetal Programming

The perinatal period represents a critical window of neurodevelopment characterized by high plasticity, during which environmental exposures can induce long-term biological programming. The fetal programming hypothesis proposes that adverse intrauterine environments, including excess glucocorticoid exposure, permanently alter physiological systems. Prenatal stress affects HPA axis regulation, GR sensitivity, and neurochemical development, increasing vulnerability to later somatic and psychiatric disorders through gene–environment interactions [42]. CRH and GCs regulate neurogenesis and brain maturation; however, excessive exposure is associated with structural and functional brain alterations. Neurosteroids may exert protective effects in late gestation, whereas prenatal stress reduces neurosteroid signaling and impairs myelination in animal models [106]. Overall, prenatal stress during critical developmental periods may result in persistent and potentially irreversible alterations in brain development and stress regulation [107]. Importantly, emerging evidence suggests that postnatal environmental factors may partially mitigate or reverse some of these programmed effects, highlighting the continued plasticity of developmental systems.

Emerging evidence suggests that the effects of ELS may differ between males and females. Sex-specific differences have been reported in HPA axis reactivity, placental gene expression, glucocorticoid sensitivity, and neurodevelopmental outcomes following prenatal stress exposure. Experimental and clinical studies indicate that male fetuses may be more vulnerable to adverse intrauterine conditions and neurodevelopmental disruption, whereas females may exhibit greater adaptive placental responses but increased susceptibility to certain affective disorders later in life [71]. These differences are thought to reflect interactions among sex hormones, placental regulatory mechanisms, genetic factors, and developmental timing. However, findings remain heterogeneous, and further longitudinal research is required to clarify the biological mechanisms underlying sex-dependent stress responses.

## 8. Paternal Stress

Sperm function is essential for fertilization and for the transmission of paternal genetic material to the oocyte. Evidence increasingly indicates that paternal environmental exposures and lifestyle factors can induce epigenetic modifications in sperm, influencing embryonic development and offspring health. Sperm DNA integrity is a key determinant of normal embryogenesis, while epigenetic mechanisms provide a pathway for the intergenerational transmission of environmental influences [108].

Intergenerational epigenetic inheritance refers to the transmission of environmentally induced epigenetic information via the germline. In this context, preconception paternal stress may modify germ cell programming and affect developmental trajectories across generations [109,110]. Environmental stressors, including endocrine-disrupting chemicals, have been associated with impaired sperm quality and increased disease susceptibility in offspring [111]. In males, stress exposure across the lifespan—particularly during adolescence and early adulthood—may induce persistent epigenetic alterations during germ cell maturation [112]. Although mature spermatozoa are transcriptionally inactive due to histone-to-protamine replacement, emerging evidence suggests that sperm epigenetic marks remain sensitive to environmental and physiological stress, particularly during epididymal maturation. Experimental studies indicate that paternal exposure to chronic stress, metabolic challenges, and psychosocial adversity can induce epigenetic reprogramming in germ cells, with consequences for offspring phenotype. The timing of exposure appears critical, as distinct stages of spermatogenesis show differential vulnerability to stress-related modulation.

Preclinical studies demonstrate that paternal preconception stress can alter HPA axis regulation and behavioral outcomes in offspring. In rodent models, chronic paternal stress has been associated with reduced stress responsivity, altered expression of glucocorticoid-related genes, and changes in synaptic plasticity [113]. These effects may extend across generations, with evidence suggesting sex-dependent patterns of transmission. Human and animal data further support associations between paternal stress and neurodevelopmental outcomes in offspring, including impaired memory, altered stress reactivity, and affective dysregulation [114,115]. Early life paternal adversity has been linked to long-term changes in offspring stress physiology and emotional regulation [116,117]. In humans, intergenerational effects have been reported in cohorts exposed to severe trauma. The offspring of Holocaust survivors, for example, exhibit altered HPA axis function, including changes in cortisol levels and GR sensitivity, alongside increased rates of psychiatric disorders such as PTSD and depression [118]. These findings support the hypothesis that paternal trauma exposure may exert long-term biological effects across generations. Importantly, the timing of paternal stress exposure influences outcomes, with early life adversity producing distinct effects compared with adult exposure [119].

**Figure 3:**
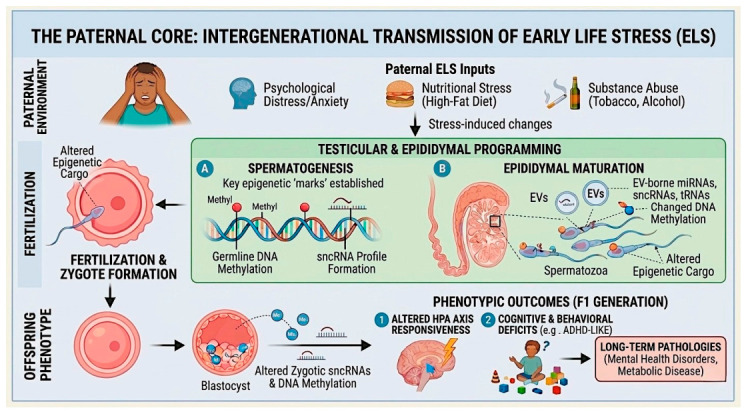
The paternal core: intergenerational transmission of early life stress (ELS).

Epidemiological studies indicate that a relevant proportion of fathers experience psychological distress during the perinatal period, with prevalence estimates ranging from 4% to 16% prenatally and 2% to 18% postnatally [120]. Contributing factors include adjustment to the paternal role, fear of childbirth, reduced perceived parenting competence, fatigue, and emotional strain associated with the transition to parenthood. Paternal stress during this period may adversely affect psychological well-being and family functioning, with potential implications for offspring development. Nevertheless, the majority of mechanistic evidence supporting paternal stress effects derives from animal studies. Human studies remain relatively limited and are predominantly observational, making causal inference challenging. Further longitudinal and mechanistic studies are required to establish causal pathways (Figure 3).

## 9. Epigenetics

Epigenetics refers to heritable and dynamic modifications that regulate gene expression without altering the underlying DNA sequence, thereby mediating the interaction between environmental exposures and the genome [121,122]. These modifications play a central role in shaping stress responsivity across the lifespan, particularly during sensitive developmental periods such as the prenatal and early postnatal stages [123]. Early life experiences can induce persistent epigenetic changes that influence which genes are expressed, and to what extent, ultimately contributing to long-term physiological and behavioral outcomes [24].

The epigenome comprises a set of chemical and structural chromatin modifications that regulate transcriptional activity. The principal epigenetic mechanisms include DNA methylation, post-translational histone modifications, and regulation by non-coding RNAs [124,125]. These processes are highly time-sensitive, with critical windows occurring around conception, prenatal development, and early childhood, when environmental perturbations may exert long-lasting programming effects.

### 9.1. DNA Methylation

DNA methylation is the most extensively studied epigenetic mechanism and involves the addition of a methyl group to cytosine residues within Cytosine–phosphate–Guanine (CpG) dinucleotides, typically resulting in transcriptional repression [126]. This process is catalyzed by DNA methyltransferases and is generally associated with gene silencing when occurring in promoter regions [127]. Importantly, DNA methylation is both stable and potentially reversible, allowing genes to be dynamically regulated across the lifespan.

Prenatal stress has been shown to induce both hypermethylation and hypomethylation in specific genomic regions, thereby altering gene expression patterns [128,129]. Similar modifications have been observed during childhood and adolescence, where stress exposure can selectively affect CpG islands and regulatory regions, including GR binding sites [130,131,132]. A key limitation in human studies is the reliance on peripheral tissues, which may not accurately reflect epigenetic changes occurring in the brain.

### 9.2. Histone Modifications and Non-Coding RNAs

Histone modifications, including acetylation and methylation, influence chromatin structure and the accessibility of transcriptional machinery. Acetylation generally promotes gene expression, whereas methylation can either activate or repress transcription depending on the context [133].

Non-coding RNAs, particularly microRNAs (miRNAs), represent an additional regulatory layer by modulating post-transcriptional gene expression. Evidence suggests that stress can alter miRNA profiles, including those present in sperm, thereby contributing to transgenerational epigenetic inheritance [113]. In animal models, specific stress-associated sperm miRNAs have been implicated in the modulation of offspring stress responses following fertilization. The relevance of these findings to human populations remains under investigation.

### 9.3. Key Stress-Related Genes and Pathways

A growing body of evidence highlights a set of stress-responsive genes that are particularly sensitive to epigenetic regulation, especially in the context of prenatal stress (Table 3).

The GR gene NR3C1 is among the most extensively studied targets. Increased DNA methylation at exon 1F of NR3C1 has been consistently associated with prenatal maternal stress and adverse psychosocial exposures, leading to altered infant cortisol reactivity and neurobehavioral outcomes [134,135]. These findings support the notion that early environmental signals can shape HPA axis regulation via epigenetic programming. Experimental studies further demonstrate that variations in maternal care can alter hippocampal NR3C1 expression through methylation-dependent mechanisms involving NGFI-A binding [136,137,138].

Similarly, the co-chaperone FK506-binding protein 5 (FKBP5) gene, which regulates GR sensitivity, has been implicated in stress-related epigenetic programming. Altered FKBP5 methylation has been associated with early life adversity, maternal stress, and increased risk for stress-related psychopathology, including PTSD [139,140,141]. These modifications may enhance HPA axis reactivity and disrupt stress regulation.

Additional genes implicated in neurodevelopmental and stress-related pathways include Brain-Derived Neurotrophic Factor (BDNF), a key neurotrophin involved in synaptic plasticity, where altered methylation has been linked to prenatal maternal depression [21,142], and the serotonin transporter gene (SLC6A4), which regulates serotonin transport and has been associated with maternal depressive symptoms during pregnancy [143].

Placental genes such as 11β-HSD2, which modulate fetal exposure to GCs, are also epigenetically regulated. Increased methylation of 11β-HSD2 has been associated with reduced enzyme expression, lower birth weight, and altered neonatal neurobehavior [144,145,146].

Moreover, genes such as CRH, AVP, and pro-opiomelanocortin (POMC), all central to HPA axis function, exhibit epigenetic alterations following ELS, contributing to the long-term dysregulation of stress responses [3,147,148].

Emerging evidence also implicates mitochondrial genes (e.g., MT-ND2) and neuronal migration-related genes such as Reelin, further underscoring the broad impact of epigenetic programming on neurodevelopment [149,150]. Furthermore, replication of epigenetic findings across independent cohorts remains inconsistent, partly due to small sample sizes and methodological variability.

### 9.4. Epigenetic Programming of the HPA Axis

The HPA axis represents a primary target of epigenetic modulation during early development. Epigenetic alterations in genes regulating glucocorticoid signaling can lead to reduced GR expression and impaired negative feedback sensitivity, resulting in heightened stress reactivity [3,151]. Both animal and human studies demonstrate that prenatal and ELS exposures are associated with coordinated epigenetic changes across multiple HPA-related genes, including NR3C1, FKBP5, and CRH [147,148].

### 9.5. Maternal and Paternal Contributions

Both maternal and paternal stress exposures have been associated with epigenetic modifications in germ cells, raising the possibility of intergenerational transmission of stress-related phenotypes [152]. However, evidence for true transgenerational inheritance in humans remains limited and requires further investigation. Environmental stressors, including psychosocial adversity and lifestyle factors, can alter epigenetic marks in sperm and oocytes, thereby influencing offspring development. Notably, paternal stress has been linked to alterations in sperm miRNA content and subsequent offspring stress responsivity [113]. The extent to which these epigenetic modifications are stable and transmissible across generations in humans remains an area of ongoing investigation.

Although accumulating evidence supports a role for epigenetic mechanisms in mediating the biological effects of ELS, many findings—particularly those related to germline transmission and transgenerational inheritance—are derived predominantly from experimental animal models. In humans, causal evidence remains limited, and the stability, persistence, and transmissibility of stress-related epigenetic modifications across generations remain areas of active investigation.

## 10. Clinical Implications and Translational Relevance

The translation of ELS research into clinical practice requires a clear distinction between biomarkers that are currently feasible for clinical use and those that remain primarily at the research stage. At present, several indicators can be considered clinically accessible or indirectly applicable in routine pediatric and mental health settings. These include psychosocial risk screening tools for ACEs, such as structured questionnaires used in primary care settings (e.g., Pediatric ACEs and Related Life-events Screener—PEARLS) [153], as well as validated behavioral and developmental assessments, including the Strengths and Difficulties Questionnaire (SDQ) [154] and the Child Behavior Checklist (CBCL) [155]. These tools may support early identification of psychosocial risk during well-child visits and routine developmental surveillance. In addition, physiological proxies such as HRV may provide indirect insight into autonomic regulation in children. Cortisol measurement (salivary or hair cortisol) is increasingly used in research settings but is not yet part of standard clinical screening due to methodological variability and the lack of standardized thresholds.

In contrast, several promising biomarkers remain primarily experimental. These include DNA methylation signatures (e.g., NR3C1, FKBP5), placental markers such as 11β-HSD2 expression, circulating inflammatory cytokine profiles as diagnostic tools, and microbiome-based signatures. While these biomarkers show strong mechanistic relevance, their clinical application is currently limited by issues of reproducibility, tissue specificity, cost, and the lack of validated reference ranges.

From a practical clinical perspective, pediatricians, therapists, psychologists, and public health professionals should prioritize the early identification of psychosocial adversity through structured screening, careful developmental surveillance, and multidisciplinary assessment. Evidence-based interventions, such as trauma-focused cognitive behavioral therapy (TF-CBT), represent first-line psychological treatment approaches for children and adolescents exposed to trauma, with demonstrated benefits in reducing post-traumatic symptoms and improving emotional regulation [156,157,158]. Additional caregiver-focused and family-based interventions may further enhance resilience and developmental outcomes [159,160].

Integration of psychosocial history with neurodevelopmental and behavioral evaluation remains the most reliable and immediately applicable approach. Preventive strategies should focus on strengthening caregiver support, reducing exposure to chronic stressors, and facilitating early referral pathways for at-risk families. Multidisciplinary collaboration between pediatricians, therapists, child psychologists/psychiatrists, and endocrinologists may be particularly important in complex cases involving neuroendocrine or growth-related manifestations.

Overall, while the field of stress-related biomarker research is rapidly evolving, most molecular and epigenetic markers remain investigational and should not yet be considered for routine clinical decision-making.

## 11. Conclusions

Contemporary lifestyles increasingly expose individuals to chronic and acute stressors, which influence daily functioning, health status, and long-term disease risk. Stress represents a fundamental neuroendocrine adaptation mechanism essential for survival; however, when dysregulated or excessive, it contributes to a broad spectrum of pathological outcomes.

Among the most influential forms of stress exposure are early life adverse experiences and childhood trauma, which interfere with neurodevelopmental programming and induce long-lasting alterations across neuroendocrine, immune, cardiovascular, metabolic, and socioemotional systems. These effects involve structural, functional, and epigenetic modifications in both the developing brain and peripheral organs. The outcomes of early stress exposure range from adaptive resilience to maladaptive trajectories associated with somatic and psychiatric disorders later in life.

ELS represents a major developmental risk factor capable of influencing multiple interconnected biological systems, including neuroendocrine, autonomic, immune, metabolic, and neurodevelopmental pathways. Through the actions of stress mediators such as glucocorticoids and catecholamines, ELS may alter developmental programming during sensitive periods of life, contributing to long-term changes in physiological regulation and health outcomes. Evidence from both human and animal studies suggests that maternal and paternal stress exposures can affect offspring development through interacting hormonal, immune, placental, and epigenetic mechanisms, thereby influencing vulnerability to a wide range of physical and mental health conditions. These effects may contribute to alterations in brain maturation, stress responsivity, immune function, and disease susceptibility across the lifespan, although their magnitude and persistence are influenced by developmental timing, environmental context, and individual susceptibility. Epigenetic processes are considered important mediators of these associations, providing a potential biological link between early environmental exposures and long-term developmental trajectories.

Although substantial progress has been made in identifying the neurobiological mechanisms linking ELS to later outcomes, the precise developmental trajectories and interactional pathways remain incompletely understood. Future research should focus on multi-level interactions between stress-related systems, gene–environment dynamics, and brain development, while prioritizing longitudinal approaches integrating neuroendocrine, epigenetic, and environmental data. Particular emphasis should be placed on refining our understanding of epigenetic mechanisms, identifying reliable biomarkers for early detection, and clarifying the biological pathways underlying stress-related vulnerability across the lifespan and potentially across generations.

Advances in this field should also inform the development of improved screening strategies and predictive biomarkers, enabling the early identification of individuals at risk and supporting timely, evidence-based preventive and therapeutic interventions. From a public health perspective, effective health promotion should include support for healthy parental lifestyles, stress management, strengthened family systems, positive parenting practices, and the prevention of all forms of family and social violence.

Early identification of childhood trauma and timely diagnosis of associated disorders are critical priorities for healthcare professionals. A multidisciplinary approach is essential not only for treatment but also as a foundational framework for effective intervention. Strengthening child resilience, family support systems, and community-based protective factors represents a key strategy for mitigating the long-term consequences of early adversity.

Although the evidence in this review was organized into distinct thematic domains to facilitate interpretation, these categories should not be viewed as independent biological entities. Rather, neuroendocrine, immune, placental, microbiome-related, developmental, and epigenetic processes interact continuously across sensitive developmental periods, collectively shaping developmental trajectories and health outcomes. The biological consequences of ELS are therefore best understood within an integrated developmental framework, in which multiple pathways converge and influence one another over time. Such interactions may contribute to more complex neurodevelopmental and health outcomes than would be predicted by any single mechanism alone.

This review should be interpreted in the context of the inherent limitations of narrative reviews. Unlike systematic reviews and meta-analyses, narrative reviews do not employ a predefined protocol, formal quality assessment, or quantitative evidence synthesis. Consequently, study selection and interpretation may be influenced by author judgment, potentially introducing selection bias and subjectivity. In addition, the reviewed literature is characterized by substantial heterogeneity in study designs, populations, stress exposure measures, and outcome assessment methods, which may limit direct comparisons across studies. Nevertheless, the narrative approach was considered appropriate for integrating evidence across multiple interconnected biological domains and developmental stages, allowing for a broader conceptual understanding of the mechanisms linking ELS with long-term health outcomes.

Several limitations of the current evidence base should be acknowledged. A substantial proportion of human studies examining ELS are observational in nature, limiting causal inference and increasing susceptibility to residual confounding. Socioeconomic disadvantage, family environment, parental mental health, genetic susceptibility, and gene–environment interactions may independently influence both exposure to adversity and later health outcomes. In addition, considerable heterogeneity exists across studies regarding the definition, timing, severity, and assessment of stress exposure, as well as the biological markers and outcome measures examined. Publication bias and the preferential reporting of significant findings may further influence the available literature. These limitations should be considered when interpreting associations between ELS, biological mechanisms, and developmental outcomes.

In conclusion, ELS and trauma represent integrated biopsychosocial experiences that affect both mind and body. Translating advances in neuroendocrine, developmental, and epigenetic research into pediatric clinical practice will be essential for improving early identification, prevention, and intervention strategies aimed at reducing the long-term burden of stress-related disorders. Given the profound and enduring effects of early adversity across the lifespan, prevention, early detection, and timely multidisciplinary intervention remain the most effective approaches for promoting lifelong health and developmental well-being.

## Figures and Tables

**Table 1:** Reported patterns of HPA axis dysregulation following early life stress.

Pattern	Typical Findings	Potential Explanatory Factors
**Hyperactivation**	Increased basal cortisol, elevated cortisol awakening response, increased ACTH reactivity	Recent stress exposure, early developmental stages, acute/chronic adversity, heightened stress sensitivity
**Hypoactivation**	Blunted cortisol response, reduced basal cortisol, attenuated stress reactivity	Severe chronic adversity, prolonged HPA axis activation leading to adaptive downregulation, PTSD-related phenotypes
**Mixed/Variable Findings**	Both hyper- and hypo-responsiveness reported across studies	Differences in age at assessment, sex, timing of exposure, type of adversity, cumulative stress burden, cortisol sampling methodology

**Table 2:** Representative biological pathways and health outcomes associated with early life stress.

Affected System/Domain	Key Pathophysiological Mechanism	Specific Health Outcomes
**Neuroendocrine**	Permanent HPA axis reprogramming; chronically elevated or blunted cortisol levels	Dysregulated stress response, hormonal imbalances, metabolic syndrome
**Neurodevelopmental**	Altered brain morphology (reduced hippocampal volume, enlarged amygdala)	ADHD, ASD, learning disabilities
**Psychiatric**	Epigenetic “scars” affecting neurotransmitter systems (GABA, Serotonin)	Major Depressive Disorder (MDD), generalized anxiety, suicide ideation
**Cardiometabolic**	Prenatal “thrifty phenotype” programming; increased sympathetic activity	Hypertension, insulin resistance, obesity, early-onset cardiovascular disease
**Immunological**	Chronic low-grade inflammation (increased IL-6, CRP); immune cell senescence	Atopic diseases (asthma, eczema), autoimmune disorders, reduced vaccine response
**Biological Aging**	Oxidative stress and ELS-induced telomere shortening	Accelerated biological aging and reduced lifespan (premature cellular aging)

**Table 3:** Detailed epigenetic and molecular targets of ELS.

Molecular Target	Epigenetic Mechanism	Impact on HPA Axis/Brain	Clinical Phenotype
**NR3C1 (GR Gene)**	Hypermethylation of Promoter Region (Exon 1F)	Downregulation of GR in the hippocampus; impaired negative feedback	Hypersensitivity to stress, emotional instability, and depression
**FKBP5**	Demethylation of Intron 7/Genetic Polymorphisms	Resistance to GCs; prolonged HPA axis activation after a stressor	Increased risk for PTSD, ADHD-like behaviors, and mood disorders
**11β-HSD2**	ELS-induced Downregulation (Placental)	Failure to deactivate maternal cortisol; “Flooding” of the fetal environment	Intrauterine Growth Restriction (IUGR), low birth weight, and future HPA priming
**SLC6A4 (5-HTT)**	Promoter Hypermethylation	Reduced serotonin transporter expression; altered amygdala reactivity	Anxiety disorders, impaired social behavior, and ASD-related traits
**BDNF**	DNA Methylation Changes in Brain-Derived Neurotrophic Factor	Reduced neuroplasticity and dendritic density in the prefrontal cortex	Cognitive deficits, memory impairment, and neurodevelopmental delays

## Data Availability

No new data were created or analyzed in this study.

## Abbreviations

The following abbreviations are used in this manuscript:

ELS	Early Life Stress
ACEs	Adverse Childhood Experiences
HPA	Hypothalamic–Pituitary–Adrenal
ANS	Autonomic Nervous System
PVN	Paraventricular Nucleus
CRH	Corticotropin-Releasing Hormone
AVP	Arginine Vasopressin
ACTH	Adrenocorticotropic Hormone
LC	Locus Coeruleus
GCs	Glucocorticoids
DHEA	Dehydroepiandrosterone
GR	Glucocorticoid Receptors
MR HRV	Mineralocorticoid Receptors Heart Rate Variability
HPT	Hypothalamic–Pituitary–Thyroid
TRH	Thyrotropin-Releasing Hormone
TSH	Thyroid-Stimulating Hormone
IL-6	Interleukin-6
TNF-α	Tumor Necrosis Factor-alpha
CRP	C-Reactive Protein
ASD	Autism Spectrum Disorder
ADHD	Attention-Deficit/Hyperactivity Disorder
PTSD	Post-Traumatic Stress Disorder
IL-1β	Interleukin-1β
IL-10	Interleukin-10
CNS	Central Nervous System
11β-HSD2	11β-hydroxysteroid dehydrogenase type 2
CpG	Cytosine–phosphate–Guanine
FKBP5	FK506-binding protein 5
BDNF	Brain-Derived Neurotrophic Factor
SLC6A4	Serotonin transporter gene
POMC	Pro-opiomelanocortin
miRNAs	microRNAs
PEARLS	Pediatric ACEs and Related Life-events Screener
SDQ	Strengths and Difficulties Questionnaire
CBCL	Child Behavior Checklist
TF-CBT	Trauma-focused cognitive behavioral therapy

## References

[1] Chrousos G.P., Gold P.W. (1992). The concepts of stress and stress system disorders: Overview of physical and behavioral homeostasis. JAMA.

[2] Chrousos G.P. (2009). Stress and disorders of the stress system. Nat. Rev. Endocrinol..

[3] Kazakou P., Nicolaides N.C., Chrousos G.P. (2022). Basic concepts and hormonal regulators of the stress system. Horm. Res. Paediatr..

[4] Chrousos G.P. (1992). Regulation and dysregulation of the hypothalamic-pituitary-adrenal axis: The corticotropin-releasing hormone perspective. Endocrinol. Metab. Clin. N. Am..

[5] Tsigos C., Chrousos G.P. (1994). Physiology of the hypothalamic-pituitary-adrenal axis in health and dysregulation in psychiatric and autoimmune disorders. Endocrinol. Metab. Clin. N. Am..

[6] Nicolaides N.C., Chrousos G., Kino T., Feingold K.R. (2020). Glucocorticoid receptor. Endotext.

[7] Agorastos A., Chrousos G.P. (2022). The neuroendocrinology of stress: The stress-related continuum of chronic disease development. Mol. Psychiatry.

[8] Tsigos C., Kyrou I., Kassi E., Chrousos G.P., Feingold K.R. (2020). Stress: Endocrine physiology and pathophysiology. Endotext.

[9] Stefanaki C., Pervanidou P., Boschiero D., Chrousos G.P. (2018). Chronic stress and body composition disorders: Implications for health and disease. Hormones.

[10] Pervanidou P., Agorastos A., Kolaitis G., Chrousos G.P. (2017). Neuroendocrine responses to early life stress and trauma and susceptibility to disease. Eur. J. Psychotraumatol..

[11] Reynolds R.M., Labad J., Buss C., Ghaemmaghami P., Räikkönen K. (2013). Transmitting biological effects of stress in utero: Implications for mother and offspring. Psychoneuroendocrinology.

[12] Agorastos A., Pervanidou P., Chrousos G.P., Baker D.G. (2019). Developmental trajectories of early life stress and trauma: A narrative review on neurobiological aspects beyond stress system dysregulation. Front. Psychiatry.

[13] Remmers M.C.C., Reijs R.P., Hoebe C.J.P.A. (2024). Defining and distinguishing early life stress, trauma, adversity, toxic and chronic stress and allostatic load: A descriptive review. Scand. J. Public Health.

[14] Morgart K., Harrison J.N., Hoon A.H., Wilms Floet A.M. (2021). Adverse childhood experiences and developmental disabilities: Risks, resiliency, and policy. Dev. Med. Child Neurol..

[15] Nicolaides N.C., Kanaka-Gantenbein C., Pervanidou P. (2024). Developmental neuroendocrinology of early-life stress: Impact on child development and behavior. Curr. Neuropharmacol..

[16] Gkesoglou T., Pervanidou P., Bozikas V.P., Agorastos A. (2022). Neurobiology of early life traumatic stress and trauma: Prolonged neuroendocrine dysregulation as a neurodevelopmental risk factor. Psychiatriki.

[17] Zheng L., Liu P., Li X., Yan S., Qiu Y., Xu Y., Yang Y., Chen L., Li G. (2025). Association between adverse childhood experiences and mortality: A systematic review and meta-analysis. Psychiatry Res..

[18] Gao X., Li G., Yang Q. (2026). Adverse childhood experiences and psychological distress: A systematic review and meta-analysis. Asian J. Psychiatr..

[19] Huang H., Yang G., Jiang X., Wang J., Shi S., Feng N., Zhong V.W. (2026). Adverse childhood experiences, genetic predisposition, and lifestyle factors: Roles in cardiometabolic-depressive disorder multimorbidity trajectories. J. Affect. Disord..

[20] Polanska K., Krol A., Merecz-Kot D., Jurewicz J., Makowiec-Dabrowska T., Chiarotti F., Calamandrei G., Hanke W. (2017). Maternal stress during pregnancy and neurodevelopmental outcomes of children during the first 2 years of life. J. Paediatr. Child Health.

[21] Braithwaite E.C., Kundakovic M., Ramchandani P.G., Murphy S.E., Champagne F.A. (2015). Maternal prenatal depressive symptoms predict infant NR3C1 1F and BDNF IV DNA methylation. Epigenetics.

[22] Kuhlman K.R., Chiang J.J., Horn S., Bower J.E. (2017). Developmental psychoneuroendocrine and psychoneuroimmune pathways from childhood adversity to disease. Neurosci. Biobehav. Rev..

[23] Cottrell E.C., Seckl J.R. (2009). Prenatal stress, glucocorticoids and the programming of adult disease. Front. Behav. Neurosci..

[24] Agorastos A., Pervanidou P., Chrousos G.P., Kolaitis G. (2018). Early life stress and trauma: Developmental neuroendocrine aspects of prolonged stress system dysregulation. Hormones.

[25] Pervanidou P., Makris G., Chrousos G., Agorastos A. (2020). Early life stress and pediatric posttraumatic stress disorder. Brain Sci..

[26] McGowan P.O., Matthews S.G. (2018). Prenatal stress, glucocorticoids, and developmental programming of the stress response. Endocrinology.

[27] Hostinar C.E., Johnson A.E., Gunnar M.R. (2015). Early social deprivation and the social buffering of cortisol stress responses in late childhood: An experimental study. Dev. Psychol..

[28] Venn R., Sigrist C., Rudzki S., D’Cunha N.M., Buchhorn R., Koenig J., Northey J.M., Naumovski N., McKune A.J. (2025). Mental health consequences of early life stress in children: The autonomic nervous system as a potential target for early detection and intervention?. Psychoneuroendocrinology.

[29] Chubar V., Vaessen T., Noortgate W.V.D., Lutin E., Bosmans G., Bekaert B., Van Leeuwen K., Calders F., Weyn S., Bijttebier P. (2023). Mild daily stress, in interaction with NR3C1 DNA methylation levels, is linked to alterations in the HPA axis and ANS response to acute stress in early adolescents. Psychoneuroendocrinology.

[30] Eng L., Lam L. (2020). Thyroid function during the fetal and neonatal periods. NeoReviews.

[31] Cheng X., Zhang H., Guan S., Zhao Q., Shan Y. (2023). Receptor modulators associated with the hypothalamus–pituitary–thyroid axis. Front. Pharmacol..

[32] Moog N.K., Entringer S., Heim C., Wadhwa P.D., Kathmann N., Buss C. (2017). Influence of maternal thyroid hormones during gestation on fetal brain development. Neuroscience.

[33] Anifantaki F., Pervanidou P., Lambrinoudaki I., Panoulis K., Vlahos N., Eleftheriades M. (2021). Maternal prenatal stress, thyroid function and neurodevelopment of the offspring: A mini review of the literature. Front. Neurosci..

[34] Shallie P.D., Naicker T. (2019). The placenta as a window to the brain: A review on the role of placental markers in prenatal programming of neurodevelopment. Int. J. Dev. Neurosci..

[35] Sodhi M.S., Sanders-Bush E. (2004). Serotonin and brain development. Int. Rev. Neurobiol..

[36] Houwing D.J., Buwalda B., van der Zee E.A., de Boer S.F., Olivier J.D.A. (2017). The serotonin transporter and early life stress: Translational perspectives. Front. Cell Neurosci..

[37] Hanson J.L., Williams A.V., Bangasser D.A., Peña C.J. (2021). Impact of early life stress on reward circuit function and regulation. Front. Psychiatry.

[38] Vela R.M. (2014). The effect of severe stress on early brain development, attachment, and emotions: A psychoanatomical formulation. Psychiatr. Clin. N. Am..

[39] Kroll-Desrosiers A.R., Nephew B.C., Babb J.A., Guilarte-Walker Y., Moore Simas T.A., Deligiannidis K.M. (2017). Association of peripartum synthetic oxytocin administration and depressive and anxiety disorders within the first postpartum year. Depress. Anxiety.

[40] Lautarescu A., Craig M.C., Glover V. (2020). Prenatal stress: Effects on fetal and child brain development. Int. Rev. Neurobiol..

[41] Gu J., Guan H.B. (2021). Maternal psychological stress during pregnancy and risk of congenital heart disease in offspring: A systematic review and meta-analysis. J. Affect. Disord..

[42] Smith K.E., Pollak S.D. (2020). Early life stress and development: Potential mechanisms for adverse outcomes. J. Neurodev. Disord..

[43] Zhu S., Shan S., Liu W., Li S., Hou L., Huang X., Liu Y., Yi Q., Sun W., Tang K. (2022). Adverse childhood experiences and risk of diabetes: A systematic review and meta-analysis. J. Glob. Health.

[44] Danese A., Tan M. (2014). Childhood maltreatment and obesity: Systematic review and meta-analysis. Mol. Psychiatry.

[45] Rosa M.J., Lee A.G., Wright R.J. (2018). Evidence establishing a link between prenatal and early-life stress and asthma development. Curr. Opin. Allergy Clin. Immunol..

[46] Guxens M., Sonnenschein-van der Voort A.M., Tiemeier H., Hofman A., Sunyer J., de Jongste J.C., Jaddoe V.W., Duijts L. (2014). Parental psychological distress during pregnancy and wheezing in preschool children: The Generation R Study. J. Allergy Clin. Immunol..

[47] Hartwig I.R., Sly P.D., Schmidt L.A., van Lieshout R.J., Bienenstock J., Holt P.G., Arck P.C. (2014). Prenatal adverse life events increase the risk for atopic diseases in children, which is enhanced in the absence of a maternal atopic predisposition. J. Allergy Clin. Immunol..

[48] LeMoult J., Humphreys K.L., Tracy A., Hoffmeister J.A., Ip E., Gotlib I.H. (2020). Meta-analysis: Exposure to early life stress and risk for depression in childhood and adolescence. J. Am. Acad. Child Adolesc. Psychiatry.

[49] Jauhar S., Johnstone M., McKenna P.J. (2022). Schizophrenia. Lancet.

[50] Jeon D., Kim S., Lee S.K., Chu K. (2024). Chronic social stress in early life can predispose mice to antisocial maltreating behavior. Encephalitis.

[51] Raleva M. (2018). Early life stress: A key link between childhood adversity and risk of attempting suicide. Psychiatr. Danub..

[52] Shin S.H., Kim Y.K. (2023). Early life stress, neuroinflammation, and psychiatric illness of adulthood. Adv. Exp. Med. Biol..

[53] Talge N.M., Neal C., Glover V. (2007). Early Stress, Translational Research and Prevention Science Network: Fetal and Neonatal Experience on Child and Adolescent Mental Health. Antenatal maternal stress and long-term effects on child neurodevelopment: How and why?. J. Child Psychol. Psychiatry.

[54] Gui Y., Wei Q., Shi Y., Zhang Y., Shi H., Xiao X. (2024). Prenatal maternal stress, sleep quality, and neonatal birth weight: A prospective cohort study. Stress Health.

[55] Garcia-Flores V., Romero R., Furcron A.E., Levenson D., Galaz J., Zou C., Hassan S.S., Hsu C.D., Olson D., Metz G.A.S. (2020). Prenatal maternal stress causes preterm birth and affects neonatal adaptive immunity in mice. Front. Immunol..

[56] Marchetto N.M., Glynn R.A., Ferry M.L., Ostojic M., Wolff S.M., Yao R., Haussmann M.F. (2016). Prenatal stress and newborn telomere length. Am. J. Obstet. Gynecol..

[57] Entringer S., Epel E.S., Lin J., Buss C., Shahbaba B., Blackburn E.H., Simhan H.N., Wadhwa P.D. (2013). Maternal psychosocial stress during pregnancy is associated with newborn leukocyte telomere length. Am. J. Obstet. Gynecol..

[58] Hussain T., Murtaza G., Metwally E., Kalhoro D.H., Kalhoro M.S., Rahu B.A., Sahito R.G.A., Yin Y., Yang H., Chughtai M.I. (2021). The role of oxidative stress and antioxidant balance in pregnancy. Mediat. Inflamm..

[59] Makris G., Eleftheriades A., Pervanidou P. (2023). Early life stress, hormones, and neurodevelopmental disorders. Horm. Res. Paediatr..

[60] Jamil S., Raza M.L., Moradikor N., Haghipanah M. (2025). Early life stress and brain development: Neurobiological and behavioral effects of chronic stress. Prog. Brain Res..

[61] Kassotaki I., Valsamakis G., Mastorakos G., Grammatopoulos D.K. (2021). Placental CRH as a signal of pregnancy adversity and impact on fetal neurodevelopment. Front. Endocrinol..

[62] Folger A.T., Eismann E.A., Stephenson N.B., Shapiro R.A., Macaluso M., Brownrigg M.E., Gillespie R.J. (2018). Parental adverse childhood experiences and offspring development at 2 years of age. Pediatrics.

[63] Siegel D.J. (2012). The Developing Mind: How Relationships and the Brain Interact to Shape Who We Are.

[64] Fan L., Kang T. (2025). Early childhood trauma and its long-term impact on cognitive and emotional development: A systematic review and meta-analysis. Ann. Med..

[65] Cook N., Ayers S., Horsch A. (2018). Maternal posttraumatic stress disorder during the perinatal period and child outcomes: A systematic review. J. Affect. Disord..

[66] Karimov-Zwienenberg M., Symphor W., Peraud W., Décamps G. (2024). Childhood trauma, PTSD/CPTSD and chronic pain: A systematic review. PLoS ONE.

[67] Makris G., Agorastos A., Chrousos G.P., Pervanidou P. (2022). Stress system activation in children and adolescents with autism spectrum disorder. Front. Neurosci..

[68] Charmandari E., Achermann J.C., Carel J.C., Söder O., Chrousos G.P. (2012). Stress response and child health. Sci. Signal..

[69] Coussons-Read M.E., Okun M.L., Nettles C.D. (2007). Psychosocial stress increases inflammatory markers and alters cytokine production across pregnancy. Brain Behav. Immun..

[70] Zijlmans M.A., Korpela K., Riksen-Walraven J.M., de Vos W.M., de Weerth C. (2015). Maternal prenatal stress is associated with the infant intestinal microbiota. Psychoneuroendocrinology.

[71] Jašarević E., Howard C.D., Misic A.M., Beiting D.P., Bale T.L. (2017). Stress during pregnancy alters temporal and spatial dynamics of the maternal and offspring microbiome in a sex-specific manner. Sci. Rep..

[72] Bolte E.E., Moorshead D., Aagaard K.M. (2022). Maternal and early life exposures and their potential to influence development of the microbiome. Genome Med..

[73] Agorastos A., Nicolaides N.C., Bozikas V.P., Chrousos G.P., Pervanidou P. (2020). Multilevel interactions of stress and circadian system: Implications for traumatic stress. Front. Psychiatry.

[74] Yu H.J., Liu X., Yang H.G., Chen R., He Q.Q. (2022). The association of adverse childhood experiences and its subtypes with adulthood sleep problems: A systematic review and meta-analysis of cohort studies. Sleep Med..

[75] Tschetter K.E., Callahan L.B., Flynn S.A., Rahman S., Beresford T.P., Ronan P.J. (2022). Early life stress and susceptibility to addiction in adolescence. Int. Rev. Neurobiol..

[76] Lee R.S., Oswald L.M., Wand G.S. (2018). Early life stress as a predictor of co-occurring alcohol use disorder and post-traumatic stress disorder. Alcohol Res..

[77] Sanchez E.O., Bangasser D.A. (2022). The effects of early life stress on impulsivity. Neurosci. Biobehav. Rev..

[78] Herzberg M.P., Gunnar M.R. (2020). Early life stress and brain function: Activity and connectivity associated with processing emotion and reward. NeuroImage.

[79] Wyrwoll C.S., Holmes M.C. (2012). Prenatal excess glucocorticoid exposure and adult affective disorders: A role for serotonergic and catecholamine pathways. Neuroendocrinology.

[80] Charil A., Laplante D.P., Vaillancourt C., King S. (2010). Prenatal stress and brain development. Brain Res. Rev..

[81] Buss C., Davis E.P., Muftuler L.T., Head K., Sandman C.A. (2010). High pregnancy anxiety during mid-gestation is associated with decreased gray matter density in 6–9-year-old children. Psychoneuroendocrinology.

[82] Teicher M.H., Samson J.A., Anderson C.M., Ohashi K. (2016). The effects of childhood maltreatment on brain structure, function and connectivity. Nat. Rev. Neurosci..

[83] Coulon M., Nowak R., Andanson S., Petit B., Lévy F., Boissy A. (2015). Effects of prenatal stress and emotional reactivity of the mother on emotional and cognitive abilities in lambs. Dev. Psychobiol..

[84] Frodl T., O’Keane V. (2013). How does the brain deal with cumulative stress? A review with focus on developmental stress, HPA axis function and hippocampal structure in humans. Neurobiol. Dis..

[85] Johnson M., Jones E., Gliga T. (2015). Brain adaptation and alternative developmental trajectories. Dev. Psychopathol..

[86] Martins Saur A., dos Santos M.A. (2021). Risk factors associated with stress symptoms during pregnancy and postpartum: Integrative literature review. Women Health.

[87] Hsu H.C., Wickrama K.A.S. (2018). Maternal life stress and health during the first 3 years postpartum. Women Health.

[88] Fisher J., Cabral de Mello M., Patel V., Rahman A., Tran T., Holton S., Holmes W. (2012). Prevalence and determinants of common perinatal mental disorders in women in low- and lower-middle-income countries: A systematic review. Bull. World Health Organ..

[89] Yonkers K.A., Smith M.V., Forray A., Epperson C.N., Costello D., Lin H., Belanger K. (2014). Pregnant women with posttraumatic stress disorder and risk of preterm birth. JAMA Psychiatry.

[90] Kingston D., Heaman M., Fell D., Dzakpasu S., Chalmers B. (2012). Factors associated with perceived stress and stressful life events in pregnant women: Findings from the Canadian maternity experiences survey. Matern. Child Health J..

[91] Alves A.C., Cecatti J.G., Souza R.T. (2021). Resilience and stress during pregnancy: A comprehensive multidimensional approach in maternal and perinatal health. Sci. World J..

[92] Heim C.M., Entringer S., Buss C. (2019). Translating basic research knowledge on the biological embedding of early-life stress into novel approaches for the developmental programming of lifelong health. Psychoneuroendocrinology.

[93] O’Donnell K.J., Glover V., Jenkins J., Browne D., Ben-Shlomo Y., Golding J., O’Connor T.G. (2013). Prenatal maternal mood is associated with altered diurnal cortisol in adolescence. Psychoneuroendocrinology.

[94] Tollenaar M.S., Beijers R., Jansen J., Riksen-Walraven J.M., de Weerth C. (2011). Maternal prenatal stress and cortisol reactivity to stressors in human infants. Stress.

[95] Barua S., Junaid M.A. (2015). Lifestyle, pregnancy and epigenetic effects. Epigenomics.

[96] Eleftheriades A., Koulouraki S., Belegrinos A., Eleftheriades M., Pervanidou P. (2025). Maternal obesity and neurodevelopment of the offspring. Nutrients.

[97] O’Donnell K.J., Bugge Jensen A., Freeman L., Khalife N., O’Connor T.G., Glover V. (2012). Maternal prenatal anxiety and downregulation of placental 11β-HSD2. Psychoneuroendocrinology.

[98] Gur T.L., Shay L., Palkar A.V., Fisher S., Varaljay V.A., Dowd S., Bailey M.T. (2017). Prenatal stress affects placental cytokines and neurotrophins, commensal microbes, and anxiety-like behavior in adult female offspring. Brain Behav. Immun..

[99] Valsamakis G., Chrousos G., Mastorakos G. (2019). Stress, female reproduction and pregnancy. Psychoneuroendocrinology.

[100] Zhang W., Li Q., Deyssenroth M., Lambertini L., Finik J., Ham J., Huang Y., Tsuchiya K.J., Pehme P., Buthmann J. (2018). Timing of prenatal exposure to trauma and altered placental expressions of HPA axis genes and neurodevelopment genes. J. Neuroendocrinol..

[101] Glover V., O’Donnell K.J., O’Connor T.G., Fisher J. (2018). Prenatal maternal stress, fetal programming, and mechanisms underlying later psychopathology—A global perspective. Dev. Psychopathol..

[102] Monk C., Feng T., Lee S., Krupska I., Champagne F.A., Tycko B. (2016). Distress during pregnancy: Epigenetic regulation of placenta glucocorticoid-related genes and fetal neurobehavior. Am. J. Psychiatry.

[103] Wang G., Huang Y., Hu T., Zhang B., Tang Z., Yao R., Huang Y., Fan X., Ni X. (2020). Contribution of placental 11β-HSD2 to the pathogenesis of preeclampsia. FASEB J..

[104] Moog N.K., Buss C., Entringer S., Shahbaba B., Gillen D.L., Hobel C.J., Wadhwa P.D. (2016). Maternal exposure to childhood trauma is associated during pregnancy with placental-fetal stress physiology. Biol. Psychiatry.

[105] Bronson S.L., Bale T.L. (2014). Prenatal stress-induced increases in placental inflammation and offspring hyperactivity are male-specific and ameliorated by maternal anti-inflammatory treatment. Endocrinology.

[106] Hirst J.J., Cumberland A.L., Shaw J.C., Bennett G.A., Kelleher M.A., Walker D.W., Palliser H.K. (2016). Loss of neurosteroid-mediated protection following stress during fetal life. J. Steroid Biochem. Mol. Biol..

[107] Nutton G., Silburn S., Arney F., Moss B. (2011). The First Five Years: Starting Early.

[108] Chan J.C., Nugent B.M., Bale T.L. (2018). Parental advisory: Maternal and paternal stress can impact offspring neurodevelopment. Biol. Psychiatry.

[109] Rodgers A.B., Bale T.L. (2015). Germ cell origins of posttraumatic stress disorder risk: The transgenerational impact of parental stress experience. Biol. Psychiatry.

[110] Klengel T., Dias B.G., Ressler K.J. (2016). Models of intergenerational and transgenerational transmission of risk for psychopathology in mice. Neuropsychopharmacology.

[111] Xu X., Miao Z., Sun M., Wan B. (2021). Epigenetic mechanisms of paternal stress in offspring development and diseases. Int. J. Genom..

[112] Short A.K., Fennell K.A., Perreau V.M., Fox A., O’Bryan M.K., Kim J.H., Bredy T.W., Pang T.Y., Hannan A.J. (2016). Elevated paternal glucocorticoid exposure alters the small noncoding RNA profile in sperm and modifies anxiety and depressive phenotypes in the offspring. Transl. Psychiatry.

[113] Rodgers A.B., Morgan C.P., Bronson S.L., Revello S., Bale T.L. (2013). Paternal stress exposure alters sperm microRNA content and reprograms offspring HPA stress axis regulation. J. Neurosci..

[114] Bohacek J., Farinelli M., Mirante O., Steiner G., Gapp K., Coiret G., Ebeling M., Durán-Pacheco G., Iniguez A.L., Manuella F. (2015). Pathological brain plasticity and cognition in the offspring of males subjected to postnatal traumatic stress. Mol. Psychiatry.

[115] Sherin J.E., Nemeroff C.B. (2011). Post-traumatic stress disorder: The neurobiological impact of psychological trauma. Dialogues Clin. Neurosci..

[116] Franklin T.B., Russig H., Weiss I.C., Gräff J., Linder N., Michalon A., Vizi S., Mansuy I.M. (2010). Epigenetic transmission of the impact of early stress across generations. Biol. Psychiatry.

[117] Kinnally E.L., Capitanio J.P. (2015). Paternal early experiences influence infant development through non-social mechanisms in rhesus macaques. Front. Zool..

[118] Martin S. (2014). Journal Watch review of influences of maternal and paternal PTSD on epigenetic regulation of the glucocorticoid receptor gene in Holocaust survivor offspring. J. Am. Psychoanal. Assoc..

[119] Batchelor V., Pang T.Y. (2019). HPA axis regulation and stress response is subject to intergenerational modification by paternal trauma and stress. Gen. Comp. Endocrinol..

[120] Philpott L.F., Leahy-Warren P., FitzGerald S., Savage E. (2017). Stress in fathers in the perinatal period: A systematic review. Midwifery.

[121] Murgatroyd C., Wu Y., Bockmühl Y., Spengler D. (2010). Genes learn from stress: How infantile trauma programs us for depression. Epigenetics.

[122] Meaney M.J., Szyf M., Seckl J.R. (2007). Epigenetic mechanisms of perinatal programming of hypothalamic-pituitary-adrenal function and health. Trends Mol. Med..

[123] Szyf M. (2019). The epigenetics of perinatal stress. Dialogues Clin. Neurosci..

[124] Babenko O., Kovalchuk I., Metz G.A. (2012). Epigenetic programming of neurodegenerative diseases by an adverse environment. Brain Res..

[125] McGowan P.O., Sasaki A., D’Alessio A.C., Dymov S., Labonté B., Szyf M., Turecki G., Meaney M.J. (2009). Epigenetic regulation of the glucocorticoid receptor in human brain associates with childhood abuse. Nat. Neurosci..

[126] Martin C., Zhang Y. (2007). Mechanisms of epigenetic inheritance. Curr. Opin. Cell Biol..

[127] Dunn E.C., Soare T.W., Zhu Y., Simpkin A.J., Suderman M.J., Klengel T., Smith A.D.A.C., Ressler K.J., Relton C.L. (2019). Sensitive periods for the effect of childhood adversity on DNA methylation: Results from a prospective longitudinal study. Biol. Psychiatry.

[128] Mychasiuk R., Schmold N., Ilnytskyy S., Kovalchuk O., Kolb B., Gibb R. (2011). Prenatal bystander stress alters brain, behavior, and the epigenome of developing rat offspring. Dev. Neurosci..

[129] Xu L., Sun Y., Gao L., Cai Y.Y., Shi S.S. (2014). Prenatal restraint stress is associated with demethylation of corticotrophin-releasing hormone (CRH) promoter and enhances CRH transcriptional responses to stress in adolescent rats. Neurochem. Res..

[130] Doherty T.S., Forster A., Roth T.L. (2016). Global and gene-specific DNA methylation alterations in the adolescent amygdala and hippocampus in an animal model of caregiver maltreatment. Behav. Brain Res..

[131] Van der Knaap L.J., Riese H., Hudziak J.J., Verbiest M.M.P.J., Verhulst F.C., Oldehinkel A.J., Van Oort F.V.A. (2014). Glucocorticoid receptor gene (NR3C1) methylation following stressful events between birth and adolescence: The TRAILS study. Transl. Psychiatry.

[132] Pérez R.F., Santamarina P., Tejedor J.R., Urdinguio R.G., Álvarez-Pitti J., Redon P., Fernández A.F., Fraga M.F., Lurbe E. (2019). Longitudinal genome-wide DNA methylation analysis uncovers persistent early-life DNA methylation changes. J. Transl. Med..

[133] Weaver I.C. (2007). Epigenetic programming by maternal behavior and pharmacological intervention: Nature versus nurture. Epigenetics.

[134] Perroud N., Rutembesa E., Paoloni-Giacobino A., Mutabaruka J., Mutesa L., Stenz L., Malafosse A., Karege F. (2014). The Tutsi genocide and transgenerational transmission of maternal stress: Epigenetics and biology of the HPA axis. World J. Biol. Psychiatry.

[135] Conradt E., Lester B.M., Appleton A.A., Armstrong D.A., Marsit C.J. (2013). The roles of DNA methylation of NR3C1 and 11β-HSD2 and exposure to maternal mood disorder in utero on newborn neurobehavior. Epigenetics.

[136] Meaney M.J., Aitken D.H., Bodnoff S.R., Iny L.J., Tatarewicz J.E., Sapolsky R.M. (2013). Early postnatal handling alters glucocorticoid receptor concentrations in selected brain regions. Behav. Neurosci..

[137] Hellstrom I.C., Dhir S.K., Diorio J.C., Meaney M.J. (2012). Maternal licking regulates hippocampal glucocorticoid receptor transcription through a thyroid hormone-serotonin-NGFI-A signaling cascade. Phil. Trans. R. Soc. B.

[138] Weaver I.C., Cervoni N., Champagne F.A., D’Alessio A.C., Sharma S., Seckl J.R., Dymov S., Szyf M., Meaney M.J. (2004). Epigenetic programming by maternal behavior. Nat. Neurosci..

[139] Paquette A.G., Lester B.M., Koestler D.C., Lesseur C., Armstrong D.A., Marsit C.J. (2014). Placental FKBP5 genetic and epigenetic variation is associated with infant neurobehavioral outcomes in the RICHS cohort. PLoS ONE.

[140] Yehuda R., Daskalakis N.P., Bierer L.M., Bader H.N., Klengel T., Holsboer F., Binder E.B. (2016). Holocaust exposure induced intergenerational effects on FKBP5 methylation. Biol. Psychiatry.

[141] Stavrou S., Nicolaides N.C., Critselis E., Darviri C., Charmandari E., Chrousos G.P. (2017). Paediatric stress: From neuroendocrinology to contemporary disorders. Eur. J. Clin. Investig..

[142] Cao-Lei L., de Rooij S.R., King S., Matthews S.G., Metz G.A.S., Roseboom T.J., Szyf M. (2020). Prenatal stress and epigenetics. Neurosci. Biobehav. Rev..

[143] Wankerl M., Miller R., Kirschbaum C., Hennig J., Stalder T., Alexander N. (2014). Effects of genetic and early environmental risk factors for depression on serotonin transporter expression and methylation profiles. Transl. Psychiatry.

[144] Jensen Pena C., Monk C., Champagne F.A. (2012). Epigenetic effects of prenatal stress on 11β-hydroxysteroid dehydrogenase-2 in the placenta and fetal brain. PLoS ONE.

[145] Marsit C.J., Maccani M.A., Padbury J.F., Lester B.M. (2012). Placental 11β-HSD methylation is associated with newborn growth and neurobehavioral outcome. PLoS ONE.

[146] Stroud L.R., Papandonatos G.D., Parade S.H., Salisbury A.L., Phipps M.G., Lester B.M., Padbury J.F., Marsit C.J. (2016). Prenatal major depressive disorder, placenta glucocorticoid and serotonergic signaling, and infant cortisol response. Psychosom. Med..

[147] Kertes D.A., Kamin H.S., Hughes D.A., Rodney N.C., Bhatt S., Mulligan C.J. (2016). Prenatal maternal stress predicts methylation of HPA-axis genes in mothers and newborns. Child Dev..

[148] Nicolaides N.C., Kyratzi E., Lamprokostopoulou A., Chrousos G.P., Charmandari E. (2015). Stress, the stress system and glucocorticoids. Neuroimmunomodulation.

[149] Lambertini L., Chen J., Nomura Y. (2015). Mitochondrial gene expression profiles and maternal psychosocial stress in pregnancy. PLoS ONE.

[150] Palacios-García I., Lara-Vásquez A., Montiel J.F., Díaz-Véliz G.F., Sepúlveda H., Utreras E., Montecino M., González-Billault C., Aboitiz F. (2015). Prenatal stress down-regulates Reelin expression via promoter methylation. PLoS ONE.

[151] Qadir M.I., Anwer F. (2019). Epigenetic modification related to acetylation of histone and methylation of DNA in immunological disorders. Crit. Rev. Eukaryot. Gene Expr..

[152] González C.R., González B. (2021). Epigenetic reprogramming in paternal germ cells under stress. Front. Endocrinol..

[153] Ye M., Hessler D., Ford D., Benson M., Koita K., Bucci M., Long D., Harris N.B., Thakur N. (2023). Pediatric ACEs and Related Life Event Screener (PEARLS) latent domains and child health in a safety-net primary care practice. BMC Pediatr..

[154] Caro-Cañizares I., Díaz de Neira-Hernando M., Pfang B., Baca-Garcia E., Carballo J.J. (2018). The Strengths and Difficulties Questionnaire-Dysregulation Profile, Non-Suicidal Self-Injury Behaviors and the Mediating Role of Stressful Life Events. Span. J. Psychol..

[155] Loeb J., Stettler E.M., Gavila T., Stein A., Chinitz S. (2011). The Child Behavior Checklist PTSD Scale: Screening for PTSD in young children with high exposure to trauma. J. Trauma Stress.

[156] Thielemann J.F.B., Kasparik B., König J., Unterhitzenberger J., Rosner R. (2022). A systematic review and meta-analysis of trauma-focused cognitive behavioral therapy for children and adolescents. Child Abus. Negl..

[157] Cohen J.A., Deblinger E., Mannarino A.P. (2026). Trauma-Focused Cognitive Behavioral Therapy for Children and Parents. Child Adolesc. Psychiatr. Clin. N. Am..

[158] McGuire A., Steele R.G., Singh A. (2021). Systematic review on the application of Trauma-Focused Cognitive Behavioral Therapy (TF-CBT) for preschool-aged children. Clin. Child Fam. Psychol. Rev..

[159] Kaminski J.W., Robinson L.R., Hutchins H.J., Newsome K.B., Barry C.M. (2022). Evidence base review of couple- and family-based psychosocial interventions to promote infant and early childhood mental health, 2010–2019. J. Marital Fam. Ther..

[160] Goodrum N.M., Prinz R.J. (2022). Family-based prevention of child traumatic stress. Pediatr. Clin. N. Am..

